# Human Papillomavirus Vaccine Administration Trends Among Commercially Insured US Adults Aged 27-45 Years Before and After Advisory Committee on Immunization Practices Recommendation Change, 2007-2020

**DOI:** 10.1001/jamahealthforum.2022.4716

**Published:** 2022-12-16

**Authors:** Ryan Suk, Kaiping Liao, Cici X. Bauer, Catherine Basil, Meng Li

**Affiliations:** 1 Department of Management, Policy and Community Health, The University of Texas Health Science Center at Houston School of Public Health, Houston; 2 Department of Biostatistics, The University of Texas MD Anderson Cancer Center, Houston; 3 Department of Biostatistics and Data Science, The University of Texas Health Science Center at Houston School of Public Health, Houston; 4 Department of Public Health, College for Health, Community and Policy, The University of Texas at San Antonio; 5 Department of Health Services Research, The University of Texas MD Anderson Cancer Center, Houston

## Abstract

**Importance:**

In 2019, the Advisory Committee on Immunization Practices (ACIP) recommended patient-clinician shared decision-making for human papillomavirus (HPV) vaccination in adults aged 27 to 45 years. Less is known about the HPV vaccine administration trends in this age group before and after this recommendation update.

**Objective:**

To examine the association between the ACIP recommendation update and the HPV vaccine administration among US adults aged 27 to 45 years.

**Design, Setting, and Participants:**

This large commercial claim-based retrospective cohort study used the Optum Clinformatics database for validated claims from January 1, 2007, through December 31, 2020. A total of 22 600 520 US adults aged 27 to 45 years without previous HPV vaccination claims during the study and enrollment period were included.

**Main Outcomes and Measures:**

The first-appearing HPV vaccination claim per individual was defined as a new HPV vaccine administration. Interrupted time-series analyses were conducted to assess the association between the ACIP update and the quarterly vaccine administration rate change. The annual rate trends across race and ethnicity groups and the proportions of vaccination cases by sub–age groups and valent types were also estimated. Vaccine administration trends were assessed by race and ethnicity in this age group because HPV vaccination trends were found to differ by race and ethnicity in the initially eligible population.

**Results:**

Among 22 600 520 final study participants, the majority were men (50.9%) and non-Hispanic White (53.4%), and the mean (SD) age when first observed was 34.6 (5.8) years. In women, the ACIP update was associated with an immediate increase in vaccine administration rate (coefficient *β*_2_, 40.18 per 100 000 persons; *P* = .01) and an increased slope (coefficient *β*_3_, 9.62 per 100 000 persons per quarter; *P* = .03) over time postupdate. The ACIP update was only associated with an immediate increase in vaccine administration in men (coefficient *β*_2_, 27.54; *P* < .001). The annual rate trends were similar across race and ethnicity groups. Age at vaccine administration shifted over time (eg, women aged 40-45 years comprised only 4.9% of vaccinations in 2017, then 19.0% in 2019, and 22.7% in 2020). The most administered HPV vaccines in 2020 were 9 valent (women, 97.0%; men, 97.7%).

**Conclusions and Relevance:**

In this population-based cohort study, there were statistically significant increases in HPV vaccine administration in adults aged 27 to 45 years after the ACIP recommendation update. Patient-clinician shared decision-making may have been the main associated factor for this increase. Further research is warranted to explore the decision-making process in receiving HPV vaccination and to develop effective decision aids to maximize the cancer prevention benefit in this age group.

## Key Points

**Question:**

What are the trends of human papillomavirus (HPV) vaccine administration among US adults aged 27 to 45 years with commercial insurance before and after the Advisory Committee on Immunization Practices recommendation update for patient-clinician shared decision-making?

**Findings:**

In this large commercial claim-based cohort study of 22.6 million enrollees aged 27 to 45 years in the US, the HPV vaccine administration rates in both women and men showed a statistically significant increase associated with the age-expanded Advisory Committee on Immunization Practices recommendation update.

**Meaning:**

Further research is warranted to explore the decision-making process in receiving HPV vaccination to maximize the cancer prevention benefit in this age group.

## Teaser

This cohort study examines the association between the Advisory Committee on Immunization Practices recommendation update for patient-clinician shared decision-making and human papillomavirus vaccine administration among US adults aged 27 to 45 years.

## Introduction

Human papillomavirus (HPV) causes cervical, oropharyngeal, anal, penile, vaginal, and vulvar cancers and is associated with approximately 45 000 new cancer diagnoses annually in the US.^1^ Although cervical cancer incidence has been declining since the introduction of cervical cancer screening, other types of HPV-associated cancer currently do not have validated screening programs. Additionally, recent studies report that oropharyngeal cancer and anal cancer incidence rates are increasing by nearly 3% annually in the US.^2,3^

The HPV vaccine is an effective prevention against HPV-associated cancers, especially when administered before exposure to HPV types covered in the HPV vaccine.^4,5,6,7^ Therefore, the US Advisory Committee on Immunization Practices (ACIP) recommends HPV vaccination for those aged 9 to 26 years (since 2006 for women and 2011 for men).^8,9^ The ACIP guideline currently recommends routine vaccination for those ages 11 to 12 years and catch-up vaccination through age 26 years.^7^ In October 2018, the US Food and Drug Administration (FDA) expanded the approved age range for the use of the 9-valent HPV vaccine to ages 27 to 45 years in both women and men.^10^ The ACIP recommended patient-clinician shared decision-making for HPV vaccination in this newly approved age group in June 2019.^7^

Individual differences in previous exposure to HPV and clinical characteristics affect the effectiveness of the HPV vaccine in this older age group. In fact, though it is reported that HPV vaccination beyond 26 years old is not cost-effective at the population level at current prices,^11,12^ evidence supports vaccine efficacy in previously unexposed older populations.^13,14^ Therefore, some of these individuals may feel the need to get vaccinated for protection against HPV. Patient-clinician shared decision-making based on the guideline update may also have changed HPV vaccine administration in this age group, as evidence shows shared decision-making improves vaccination in general.^15,16^ Moreover, most commercial insurances cover vaccination for those recommended by the ACIP^17^; thus, there may be an expected trend change in the HPV vaccination rate in this age group after the FDA approval and the ACIP recommendation update.

However, there is a lack of evidence-based assessment of such trend change. Therefore, this cohort study aimed to determine the temporal trend of the HPV vaccine administration rate and the association between the ACIP recommendation update and the vaccine administration change among adults 27 to 45 years old who were enrolled in commercial insurance from 2007 to 2020. We also assessed if vaccine administration trends differed by race and ethnicity in this age group because HPV vaccination trends were found to differ by race and ethnicity in the initially eligible population.^18,19^ We additionally assessed how the makeup of ages when administered and valent types of the vaccine shifted over the years.

## Methods

### Data Source

For these analyses, we conducted a retrospective cohort study using the Optum Clinformatics database for validated claims from January 1, 2007, through December 31, 2020. The Clinformatics data set covers approximately 64.3 million lives enrolled in commercial health plans and comprises deidentified medical and pharmacy claims from all 50 US states. Individuals are given unique identifiers that allow researchers to follow individuals as they enroll, disenroll, and re-enroll in the health plan.^20^ Demographic information—including age, sex, and race and ethnicity—of the plan members, dates of medical service, diagnosis (*International Classification of Diseases* codes) and procedures (*Current Procedural Terminology* codes), and payment information are available.

This study was deemed exempt from review by the institutional review board of The University of Texas Health Science Center owing to use of deidentified data. This study followed the Strengthening the Reporting of Observational Studies in Epidemiology (STROBE) reporting guideline for cohort studies.

### Study Population and Variables

In this study, we investigated the vaccine administration trend, defined as the rate of having at least 1 HPV vaccination claim (the first-appearing HPV vaccination claim) during the study period among the eligible population. The study aimed to identify the general trends in the HPV vaccine administration itself among newly vaccine-approved older age groups rather than their up-to-date vaccine coverage. The total study population (the denominator) was identified as adults aged 27 to 45 years for each enrollment time period (either year or quarter) who did not have any HPV vaccination claims in the past time periods of their enrollment. For example, if an individual had the first-appearing HPV vaccination claims in 2010, we excluded the individual from the denominator in calculating the HPV vaccination rate in 2011 and thereafter. We identified the administration of the HPV vaccine by *Current Procedural Terminology* codes in the medical claims file: 90649 for 4 valent, 90650 for 2 valent, or 90651 for 9 valent. We additionally identified individuals’ race and ethnicity and sex. In the Clinformatics data, race and ethnicity are defined as Asian, Hispanic, non-Hispanic Black, non-Hispanic White, and unknown, and are derived using public records and self-reported surveys, then an algorithm based on the enrollee’s zip code with their first, middle, and last names (E-Tech 7.3 [Ethnic Technologies]).^21,22^ This algorithm has demonstrated 97% specificity, 48% sensitivity, and 71% positive predictive value for estimating the race of Black individuals.^23^ The sex variable is categorized as binary (men, women).

### Statistical Analysis

Descriptive statistics were used to summarize the characteristics of the final study population of adults aged 27 to 45 years. To quantify exploratory trends in annual administration rates and determine any trend change in the study period (ie, joinpoint) by sex and race and ethnicity, we used the joinpoint regressions.^24^ To assess the shifts in the distribution of age at administration in these older adults, we calculated the proportions of sub–age groups by year (27-30, 31-34, 35-39, and 40-45 years) among the vaccinated participants. We also calculated the proportions of valent types of HPV vaccine administered per year.

Finally, we conducted an interrupted time-series analysis to assess the effect of the ACIP recommendation update.^25^ This analysis evaluates the immediate level change (difference in rates) before and after the guideline update and the postupdate trend change over time (difference in slopes). Data were grouped quarterly to test the hypothesis. We hypothesized a trend break in the second quarter of 2019 (ACIP update) for the base-case analysis. Additionally, we tested a trend break in the fourth quarter of 2018 (FDA approval) to assess if FDA approval was associated with any change. Detailed methods are explained in eMethods 1 and 2 in the Supplement.

### Sensitivity Analysis

To address several limitations of the nature of claims data, we performed 3 different sensitivity analyses using annual information. In the Clinformatics database, the enrollee’s age was calculated by subtracting birth year from the claim year, which could result in a 1-year difference (eg, 27 vs 26). Some of the 27-year-olds with vaccine claims might also have initiated the series of vaccination when they were 26 years old while enrolled in another plan. Therefore, we performed a sensitivity analysis (sensitivity analysis 1) that excluded those aged 27 years from the study sample to address these concerns. We conducted sensitivity analysis 2, where we only included the birth cohorts that were never eligible for the HPV vaccine until 2018’s new FDA approval (women born in or before 1979 and men born in or before 1984 among the ages up to 45 years every year). Because these birth cohorts were not eligible for the HPV vaccine until the new approval, they had a minimal likelihood of getting vaccinated while enrolled in another plan. For the primary analysis, we did not restrict the study samples based on the enrollment period because the main interest in this study is new HPV administration rather than up-to-date vaccination. To test the robustness of the main findings, we conducted sensitivity analysis 3, only including those fully enrolled for each calendar year.

All analyses were conducted stratified by sex. Statistical significance was assessed at *P* < .05, and all hypotheses were 2-sided. We used Joinpoint Trend Analysis software, version 4.9.0.1 (National Cancer Institute); SAS, version 9.4 (SAS Institute); and Stata, version 17.0 (StataCorp) for all analyses.

## Results

Among 22 600 520 final study participants (65 043 302 person-years), 50.9% were men and 53.4% were non-Hispanic White; 18.6% of study participants had missing information on race and ethnicity. The mean (SD) age when first observed was 34.6 (5.8) years (Table 1). Between 2007 and 2020, 9819 men and 35 039 women were identified as having at least 1 HPV vaccination claim during the study period.

**Table 1.** Univariate Characteristics of the Total Study Sample of Adults Aged 27-45 Years

Characteristic	No. (%)
Total	22 600 520 (100)
Person-years^a^	65 043 302
Age when first observed, mean (SD), y	34.6 (5.8)
Sex	
Women	11 092 932 (49.1)
Men	11 507 588 (50.9)
Race and ethnicity^b^	
Asian	1 342 032 (5.9)
Hispanic	2 843 569 (12.6)
Non-Hispanic Black	2 148 299 (9.5)
Non-Hispanic White	12 067 056 (53.4)
Unknown	4 199 564 (18.6)
First year observed in the data^c^	
2007	5 479 387 (24.2)
2008	1 529 999 (6.8)
2009	1 221 144 (5.4)
2010	1 150 257 (5.1)
2011	1 111 205 (4.9)
2012	1 094 653 (4.8)
2013	1 159 470 (5.1)
2014	1 170 043 (5.2)
2015	1 570 057 (7.0)
2016	1 667 725 (7.4)
2017	1 557 576 (6.9)
2018	1 453 099 (6.4)
2019	1 319 172 (5.8)
2020	1 116 733 (4.9)

^a^ Person-years were calculated to describe the study sample’s baseline characteristics.

^b^ In the Optum Clinformatics database, race and ethnicity data are derived using public records and self-reported surveys, then an algorithm based on the enrollee’s zip code with their first, middle, and last names.

^c^ The number and the proportion of enrollees that were observed in the claims data enrollment file for the first time by calendar year.

**Figure 1.**
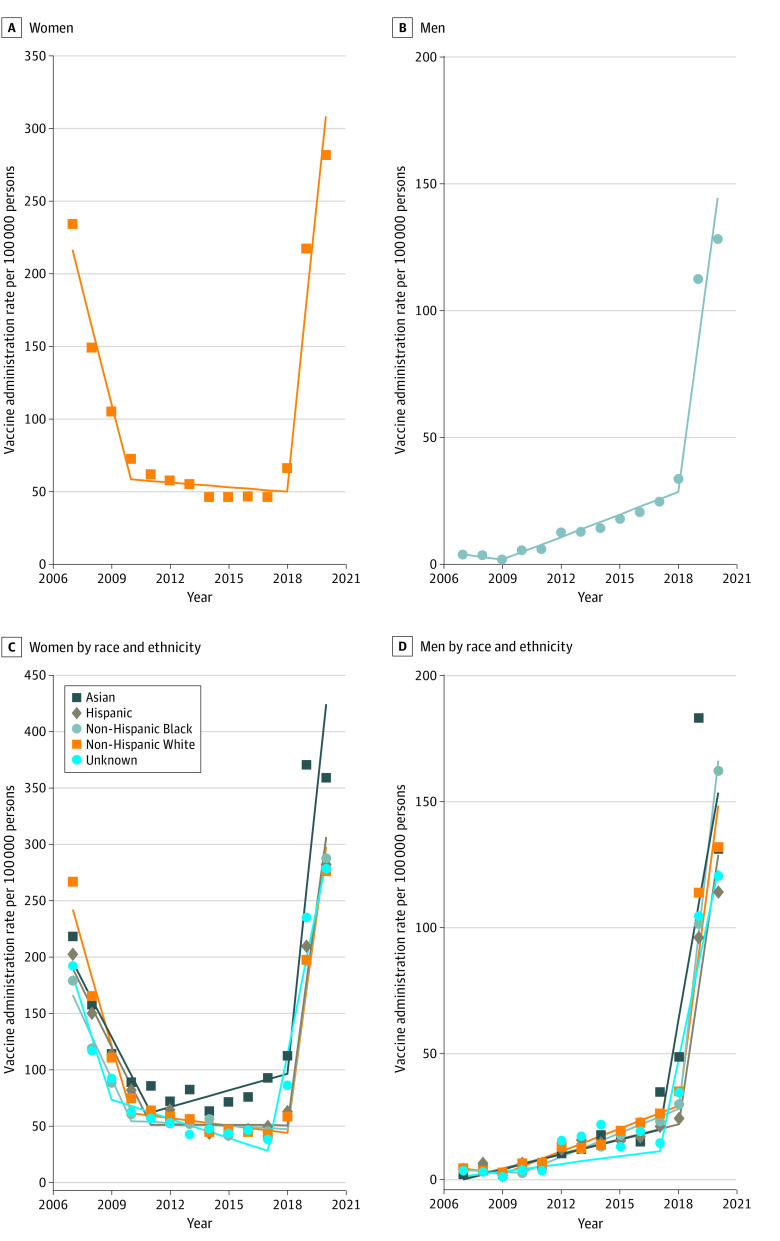
Temporal Trends of Annual Human Papillomavirus Vaccine Administration Rates in US Adults Aged 27-45 Years by Sex and Race and Ethnicity, 2007-2020

Figure 1A and B and eTable 1 in the Supplement show the sex-specific temporal trends of annual HPV vaccine administration rates (first-appearing vaccine claim per 100 000 persons). In women, the rate in this age group declined between 2007 and 2010 (slope, −52.6 per 100 000 person rate decrease per year; *P* < .001), then became stagnant until 2018 (slope, −1.1; *P* = .32). After 2018, there was a statistically significant increase in the administration rate (slope, 129.3; *P* < .001). The administration rates were consistently low for men, with no change from 2007 until 2009 (slope, −1.2; *P* = .31). From 2010, it increased by 3.0 per 100 000 persons annually until 2018; after 2018, there was a statistically significant increase in the rate, as in women (slope, 57.7; *P* < .001). There were similar patterns across the race and ethnicity groups in both men and women (Figure 1C and D and eTable 2 in the Supplement).

**Figure 2.**
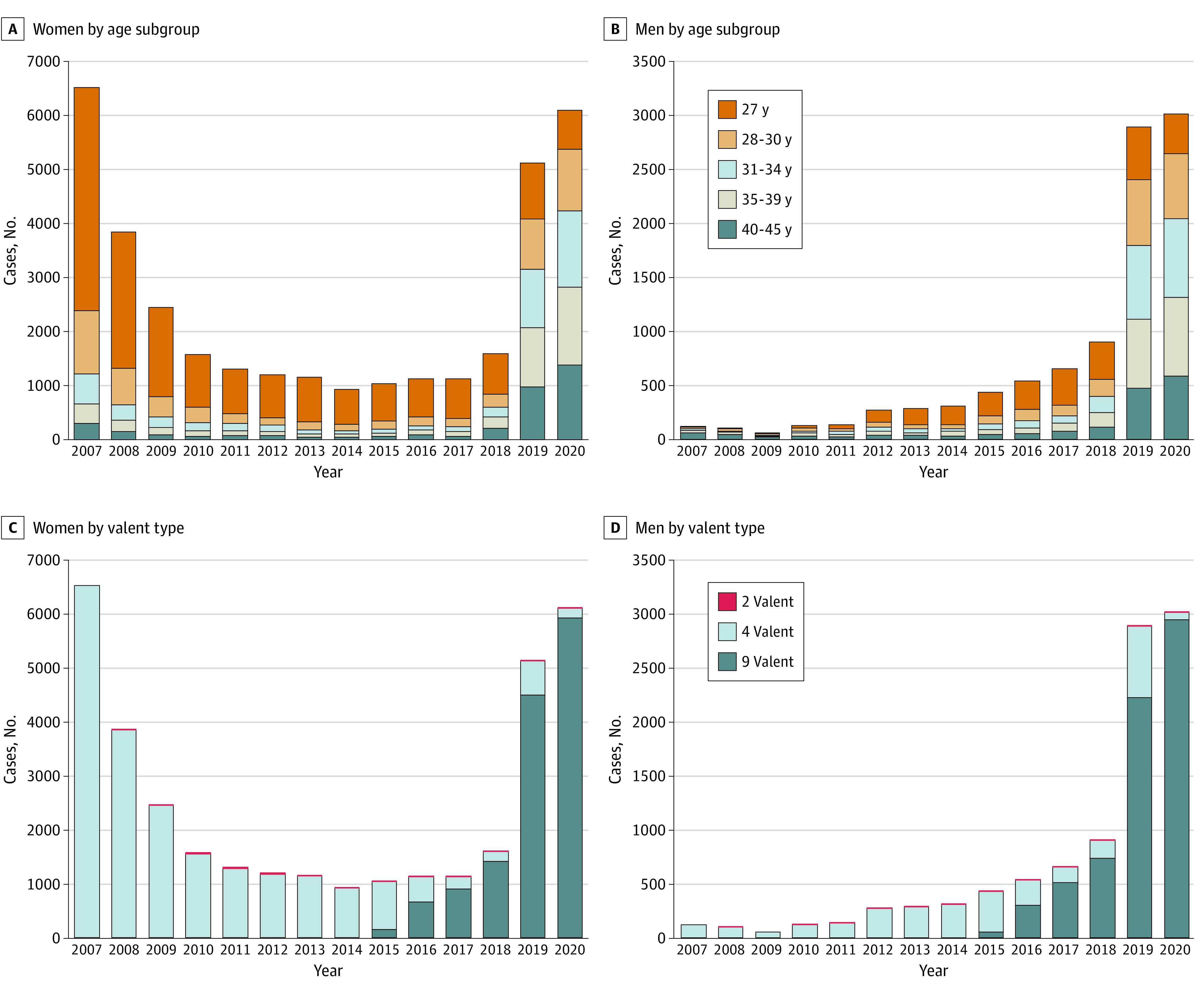
Distribution of Annual Human Papillomavirus Vaccines Among Men and Women by Age and Valent Type, 2007-2020

Age-stratified analysis showed a shift in age distribution among the vaccinated individuals over time (Figure 2A and B and eTable 3 in the Supplement). In women, before the ACIP recommendation change (2007-2018), those aged 27 years made up the majority (ranging 62.1%-71.4% in 2007-2017 and 47.2% in 2018). After 2018, women aged 31 to 45 years comprised the majority of the HPV vaccination cases (61.4% in 2019 and 69.4% in 2020). Those aged 27 years accounted for only 20.3% of vaccinations in 2019 and 11.9% in 2020. On the other hand, those in the oldest age group (40-45 years) made up only 4.9% in 2017, but they comprised 19.0% in 2019 and 22.7% in 2020. Similar patterns were observed in men; those aged 27 to 30 years accounted for the majority of vaccine administrations between 2011 and 2018 (when men were recommended for HPV vaccination), then ages 31 to 45 years made up the majority in 2019 (62.2%) and 2020 (67.7%).

The proportion of each valent type used for annual HPV vaccine cases was also identified (Figure 2C and D and eTable 4 in the Supplement). The 9-valent HPV vaccine administration was introduced in the data in 2015, and its case increased thereafter (eg, among women, 13.9% in 2015 vs 97.0% in 2020). On the other hand, the 4-valent vaccine comprised the most cases until 2014 (with a very small portion of 2 valent), and then its use declined over the years (eg, among women, 85.4% in 2015 vs 2.9% in 2020).

Results from interrupted time-series analyses using quarterly data showed that the ACIP update was associated with improved HPV vaccine administration rates (Table 2 and eFigure in the Supplement). In women, the ACIP update was associated with an immediate increase in vaccine administration (coefficient *β*_2_, 40.18 per 100 000 persons; *P* = .01) and an increased slope over time postupdate (coefficient *β*_3_, 9.62 per 100 000 persons per quarter; *P* = .03). The ACIP update was only associated with an immediate increase in vaccine administration in men (coefficient *β*_2_, 27.54; *P* < .001). When using the FDA approval as a trend break, it was associated with a slope increase in women (coefficient *β*_3_, 10.06; *P* < .001) but both an immediate level increase (coefficient *β*_2_, 9.25; *P* = .04) and slope increase (coefficient *β*_3_, 3.12; *P* = .001) in men.

**Table 2.** Interrupted Time-Series Analysis of HPV Vaccine Administration Rates Among Adults Aged 27-45 Years by Sex Before and After ACIP Recommendation Update or FDA Approval^a^

Variable	Estimate of immediate change^b^		Estimate of slope change^c^	
	Coefficient *β*_2_ (95% CI)	*P* value	Coefficient *β*_3_ (95% CI)	*P* value
ACIP update				
Women	40.18 (8.85 to 71.52)	.01	9.62 (1.12 to 18.12)	.03
Men	27.54 (20.49 to 34.59)	<.001	0.16 (−1.41 to 1.73)	.84
FDA approval				
Women	22.53 (−2.33 to 47.39)	.08	10.06 (4.80 to 15.31)	<.001
Men	9.25 (0.70 to 17.80)	.04	3.12 (1.28 to 4.96)	.001

Abbreviations: ACIP, Advisory Committee on Immunization Practices; FDA, US Food and Drug Administration; HPV, human papillomavirus.

^a^ Prais-Winsten regression, which is based on the generalized least squares method accounting for serial autocorrelation, was used, with added robust standard errors. The Durbin-Watson d statistic was used to assess how the model accounted for first-order correlation. Each model included as independent variables time (in quarters) since the start of the study, an indicator variable for time occurring before or after ACIP update or FDA approval (coded 0 before and coded 1 after), and a continuous variable counting the number of quarters after ACIP update or FDA approval.

^b^ Immediate change (coefficient *β*_2_) was defined as the change in the estimated HPV administration rate (per 100 000) derived from the fitted model at the point of the ACIP update or FDA approval.

^c^ Slope change (coefficient *β*_3_) represents the difference in HPV administration rate trends before and after the ACIP update or FDA approval (difference between postupdate slope and preupdate slope).

**Figure 3.**
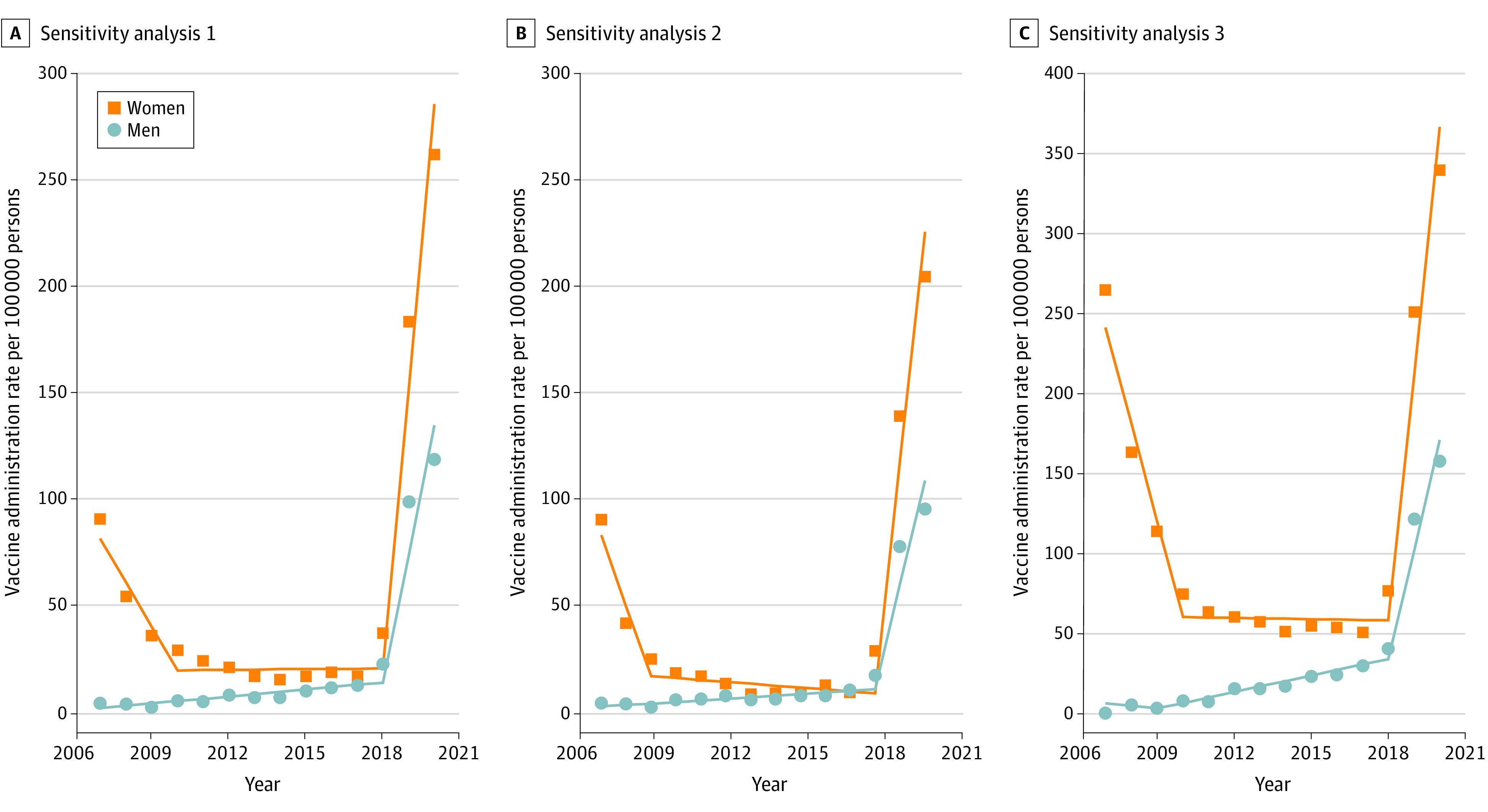
Temporal Trends of Annual Human Papillomavirus Vaccine Administration Rates Among Men and Women by Sensitivity Analysis, 2007-2020 A, Vaccine administration rates among men and women aged 28 to 45 years. B, Vaccine administration rates among birth cohorts that were not eligible in the initial recommendation (women born in or before 1979 and men born in or before 1984 among the ages up to 45 years every year). C, Vaccine administration rates among men and women fully enrolled for each calendar year.

Sensitivity analysis 1 excluded those aged 27 years (Figure 3A and eTable 5 in the Supplement) and showed a statistically significant increase in trends after 2018 that continued into 2020 and remained in both women (slope, 132.5; *P* < .001) and men (slope, 60.5; *P* < .001). Sensitivity analysis 2, which only included the birth cohorts that were never vaccine eligible until the new approval, showed similar statistically significant increasing trends after 2018 in both women (slope, 108.4; *P* = .003) and men (slope, 57.7; *P* < .001) (Figure 3B and eTable 6 in the Supplement). Sensitivity analysis 3, which only included those with full enrollment for each calendar year, also showed similar trends after 2018 in both women (slope, 154.5; *P* < .001) and men (slope, 68.5; *P* < .001), supporting the robustness in the present findings (Figure 3C and eTable 7 in the Supplement).

## Discussion

In this population-based cohort study, we found that there was a statistically significant increase in the HPV vaccine administration rate after 2018 among adults aged 27 to 45 years. Women and men showed distinct patterns before 2018, majorly due to the different timings of HPV vaccine recommendations. In this hypothesis-driven analysis, the results showed that the ACIP recommendation change was statistically significantly associated with the increase in HPV vaccine administration rate in this population.

The HPV vaccination rate among those aged 9 to 26 years showed considerable disparities across racial and ethnic groups.^18,19^ However, this study did not find statistically significant differences in administration trends among newly recommended ages. Evidence suggests racial and ethnic disparities in HPV-associated cancer incidence.^3,26,27^ Further research is needed on how catch-up vaccination in this age group can help reduce these existing disparities.

In 2019 and 2020, those aged 31 to 45 years made up the majority of the vaccinated adults aged 27 to 45 years. This makeup was the opposite until 2018, whereas the majority of the cases were among the age group of 27- to 30-year-olds. These individuals might have missed their vaccination schedule when they were still eligible and now have received catch-up vaccination as they became eligible again. On the other hand, many of these individuals aged up to 45 years who received the vaccine were never eligible for the initial ACIP HPV-vaccine recommendation (ie, women born in 1979 or before and men born in 1984 or before). Shared decision-making may have been effective for those who were never eligible but had perceived benefits of the HPV vaccine.

The dose-specific proportions can be explained by the availability of the vaccine and the ACIP recommendation. In the US, all 2-, 4-, and 9-valent types are approved for use among women, while only 4 valent and 9 valent are approved for use in men. The 4-valent HPV vaccine has been available and recommended since 2006,^28^ and as of late 2016, only the 9-valent vaccine has been distributed in the US.^7^ Because some adults without adequate vaccination might be at risk for a new HPV infection targeted by the 9-valent vaccine, HPV vaccination may benefit these individuals.

Previous studies that surveyed adults aged 27 to 45 years after the ACIP recommendation update found that the majority of these adults were likely to ask health care professionals about HPV vaccination and willing to get a vaccine if recommended by their clinician.^29,30^ The likelihood of receiving the HPV vaccine was highly associated with the perceived likelihood of benefiting from the vaccine.^29^ Similarly, according to a study that surveyed primary care physicians, the majority of physicians reported that they would be more likely to recommend HPV vaccination to adults aged 27 to 45 years due to the new ACIP recommendation.^31^ Evidence also supports the substantial effectiveness of patient-clinician shared decision-making in vaccination, cancer prevention, and other health outcomes in general.^15,16,32,33^ Especially because the HPV vaccine has been available for more than a decade, shared decision-making may benefit those interested in vaccination based on their perceived benefit but who missed their vaccination schedule or were not eligible. However, also the majority of the physician respondents were unsure of what to emphasize during the shared clinical decision-making regarding HPV vaccination.^31^ The present findings showed a statistically significant increase in HPV vaccination in this age group after the ACIP update, yet they make up very low rates. These studies together represent the great interest in HPV vaccination among both adults aged 27 to 45 years and health care professionals, as well as the potential effectiveness of their shared decision-making, though there remains a need for further research on enhancing this process (eg, decision aids).^34^

The current updated recommendation for age-expanded HPV vaccination provides an opportunity to prevent cancers in those who are at risk of new HPV infection and were not adequately vaccinated. Proper shared decision-making that allows health care professionals to discuss HPV vaccination with individuals who are most likely to benefit is key to maximizing the cancer prevention effects. This process is particularly needed to decide who should be prioritized because the universal vaccination is not cost-effective in this age group and there is a limited vaccine supply.^35^ Effective shared decision-making for selective age-expanded HPV vaccination most likely involves a conversation about the patient’s sexual history. However, according to a study in 2020, 65% of primary care visits did not involve sexual history taking.^36^ The sexual history taking based on the validated model, despite being recommended by the Centers for Disease Control and Prevention, only comprised 1% of the visits.^36^ In 2020, the US Preventive Services Task Force updated its guideline on behavioral counseling interventions for reducing sexually transmitted infections.^37^ To successfully implement this guideline, it is crucial for health care professionals to initiate a conversation about the patient’s sexual history.^38^ In addition, it is necessary that the health care professionals recognize the diversity of gender identities and sexual orientations for more effective sexual history taking^39^ and shared decision-making in HPV vaccination. Although this US Preventive Services Task Force guideline is not exclusively for HPV vaccination in older adults, it could provide an opportunity to identify those who might benefit from the age-expanded HPV vaccination.

In recognizing the challenges from the health care professionals’ side to identify the population that would most likely benefit from the age-expanded catch-up vaccination, it is equally important that patients initiate the shared decision-making. The literature indicates the patients’ awareness of age expansion, belief in vaccine effectiveness, and perceived benefit of HPV vaccination were associated with the likelihood of asking their clinicians about the HPV vaccination in this age group.^29^ Due to the information asymmetry between clinician and patient regarding patients’ sexual history and other related clinical factors, patient-initiated shared decision-making might be meaningful in age-expanded HPV vaccination. However, previous nationally representative studies found that US adults aged 27 to 45 years had a low level of awareness and knowledge about HPV-related information.^40,41^ For patient-initiated conversation about HPV vaccination, disseminating information about the recommendation update and vaccine benefits is instrumental in reaching more people who likely will benefit from vaccination.

### Limitations and Strengths

Limitations of this study include the sample of commercially insured adults, which limits the generalizability of the findings to all US adults in this age range. Due to the nature of insurance claims data, this study is also subject to limitations such as loss to follow-up (lack of utilization information before or after the enrollment) and incomplete or inaccurate administrative coding. Derivation of race and ethnicity information is also subject to limitations due to the limited validity of its method,^42^ and it warrants further research in HPV vaccine utilization across race and ethnicity among 27- to 45-year-olds. We also acknowledge the limitation of the claims data only with a binary sex variable. Gender identity, along with sexual orientation, can be an important factor in HPV vaccination with a possibly different burden of HPV.^43,44,45^

Despite these limitations, this study adds to the literature by providing an analysis of large numbers of adults in the US. Claims data are utilized often in vaccination administration studies,^46,47,48,49^ and the longitudinal information with dates of health care utilization allowed us to identify the exact timing of HPV vaccine administration and avoid biases related to self-report.

## Conclusions

This cohort study found statistically significant increases in HPV vaccine administration in adults aged 27 to 45 years after the FDA approval of use and the ACIP recommendation update. For maximizing the cancer prevention benefit in this age group, effective patient-clinician shared decision-making is crucial. Future studies are needed to address the difficulties surrounding identifying those who will most likely benefit from the vaccine and the unsureness of what to emphasize during shared decision-making. Developing effective decision aids would be the key to successfully implementing this updated ACIP guideline.

## Notes

Supplement. **eMethods 1.** Joinpoint model selection **eMethods 2.** Interrupted time series analysis **eTable 1.** Annual HPV vaccine administration rate and temporal trends among adults aged 27-45 years by sex, 2007-2020 **eTable 2.** Race and ethnicity-specific annual vaccine administration rate and temporal trends among adults aged 27-45 years by sex, 2007-2020 **eTable 3.** Age distribution of annual HPV vaccination cases among adults aged 27-45 years by sex, 2007-2020 **eTable 4.** Valent type distribution of annual HPV vaccination cases among adults aged 27-45 years by sex, 2007-2020 **eFigure.** Interrupted time series analysis of HPV vaccine administration rates among adults aged 27-45 before and after ACIP recommendation update or FDA approval **eTable 5.** Sensitivity analysis 1 (annual HPV vaccine administration rate and temporal trends among adults aged 28-45 years by sex) 2007-2020 **eTable 6.** Sensitivity analysis 2 (annual HPV vaccine administration rate and temporal trends among the birth cohorts who were not eligible before the ACIP change (ages up to 45)) **eTable 7.** Sensitivity analysis 3 (annual HPV vaccine administration rate and temporal trends among enrollees with a full year enrollment per every calendar year)
eReferences
