# Supplementary material for: Human Papillomavirus Vaccine Administration Trends Among Commercially Insured US Adults Aged 27-45 Years Before and After Advisory Committee on Immunization Practices Recommendation Change, 2007-2020
Source: JAMA Health Forum. 2022 Dec 16;3(12):e224716. doi: 10.1001/jamahealthforum.2022.4716 (PMC9856534; doi:10.1001/jamahealthforum.2022.4716)
Supplement: Supplement. — eMethods 1. Joinpoint model selection eMethods 2. Interrupted time series analysis eTable 1. Annual HPV vaccine administration rate and temporal trends among adults aged 27-45 years by sex, 2007-2020 eTable 2. Race and ethnicity-specific annual vaccine administration rate and temporal trends among adults aged 27-45 years by sex, 2007-2020 eTable 3. Age distribution of annual HPV vaccination cases among adults aged 27-45 years by sex, 2007-2020 eTable 4. Valent type distribution of annual HPV vaccination cases among adults aged 27-45 years by sex, 2007-2020 eFigure. Interrupted time series analysis of HPV vaccine administration rates among adults aged 27-45 before and after ACIP recommendation update or FDA approval eTable 5. Sensitivity analysis 1 (annual HPV vaccine administration rate and temporal trends among adults aged 28-45 years by sex) 2007-2020 eTable 6. Sensitivity analysis 2 (annual HPV vaccine administration rate and temporal trends among the birth cohorts who were not eligible before the ACIP change (ages up to 45)) eTable 7. Sensitivity analysis 3 (annual HPV vaccine administration rate and temporal trends among enrollees with a full year enrollment per every calendar year) eReferences [file jamahealthforum-e224716-s001.pdf]

Supplemental Online Content

Suk R, Liao K, Bauer CX, Basil C, Li M. Human papillomavirus vaccine administration trends among commercially insured US adults aged 27-45 years before and after Advisory Committee on Immunization Practices Recommendation change, 2007-2020. *JAMA Health Forum*. 2022;3(12):e224716. doi:10.1001/jamahealthforum.2022.4716

eMethods 1. Joinpoint model selection

eMethods 2. Interrupted time series analysis

eTable 1. Annual HPV vaccine administration rate and temporal trends among adults aged 27-45 years by sex, 2007-2020

eTable 2. Race and ethnicity-specific annual vaccine administration rate and temporal trends among adults aged 27-45 years by sex, 2007-2020

eTable 3. Age distribution of annual HPV vaccination cases among adults aged 27-45 years by sex, 2007-2020

eTable 4. Valent type distribution of annual HPV vaccination cases among adults aged 27-45 years by sex, 2007-2020

eFigure. Interrupted time series analysis of HPV vaccine administration rates among adults aged 27-45 before and after ACIP recommendation update or FDA approval

eTable 5. Sensitivity analysis 1 (annual HPV vaccine administration rate and temporal trends among adults aged 28-45 years by sex) 2007-2020

eTable 6. Sensitivity analysis 2 (annual HPV vaccine administration rate and temporal trends among the birth cohorts who were not eligible before the ACIP change (ages up to 45))

eTable 7. Sensitivity analysis 3 (annual HPV vaccine administration rate and temporal trends among enrollees with a full year enrollment per every calendar year)

eReferences

This supplemental material has been provided by the authors to give readers additional information about their work.

© 2022 Suk R et al. *JAMA Health Forum.*

eMethods 1. Joinpoint model selection

To identify the exploratory annual temporal trends and joinpoints (the calendar years where the trends change significantly), the Joinpoint program developed by the National Cancer Institute was used. The joinpoint regressions select the best-fitting piecewise linear regression model to identify calendar years when the estimated temporal slopes changed significantly, allowing for the minimum number of joinpoints necessary to fit the data. Joinpoint software performs sequential permutation tests to select the final model that indicates the significant trend changes. The permutation test was used repeatedly for testing between two different joinpoint models, a simpler model with fewer joinpoints called the null model and a more complicated model called the alternative model. The permutation test was specifically chosen because this model generally produces more conservative results and detects fewer joinpoints than other approaches, especially if the slope changes are small. The statistical details of the methodology are reported elsewhere.[1,2]

eMethods 2. Interrupted time series analysis

For the interrupted time series analysis, we used the itsa command in Stata statistical software (StataCorp, version 17.0). This command includes the adjustment for autocorrelation within time-series data, identifies temporal trends, and estimates immediate changes at the breakpoints. We hypothesized a trend break in the second quarter of 2019 (ACIP guideline update) for the base-case analysis and also conducted a sensitivity analysis for a trend break in the fourth quarter of 2018 (FDA approval). We used Prais-Winsten regression, which is based on the generalized least-squares method accounting for serial auto-correlation, and added robust standard errors. We used the Durbin-Watson d statistic to assess how the model accounted for first-order correlation. The itsa regression model assumes the following:

$$Y_t = \beta_0 + \beta_1 T_t + \beta_2 X_t + \beta_3 X_t T_t + \mathcal{E}_t$$

Y_t is the aggregated outcome variable (quarterly HPV vaccine administration rate per 100,000 persons) measured at each equally spaced time point t (by quarter), T_t is the time since the start of the study period, X_t is the dummy variable indicating the intervention status (pre- or post-policy), and $X_t T_t$ is an interaction term of time and intervention indicator. Coefficient β_0 represents the intercept of the outcome variable, and β_1 represents the slope of the pre-policy period. Coefficient β_2 represents the immediate rate change (level change) at the trend break (introduction of policy), while β_3 represents the difference between pre-policy and post-policy slopes (trend change). The statistical details of the methodology are reported elsewhere.[3]

© 2022 Suk R et al. *JAMA Health Forum.*

eTable 1. Annual HPV vaccine administration rate and temporal trends among adults aged 27-45 years by sex, 2007-2020

Women	
Year	Rate per 100,000 persons
2007	234.76
2008	149.81
2009	105.74
2010	73.07
2011	62.50
2012	58.10
2013	55.89
2014	46.68
2015	46.70
2016	47.08
2017	46.83
2018	66.76
2019	218.05
2020	282.27
Year segment	Slope (*p-value*)
2007-2010	-52.64 (*p<.001*)
2010-2018	-1.07 (*p=.32*)
2018-2020	129.30 (*p<.001*)
Men	
Year	Rate per 100,000 persons
2007	4.32
2008	3.99
2009	2.46
2010	5.89
2011	6.48
2012	13.09
2013	13.34
2014	14.71
2015	18.36
2016	21.03
2017	25.01
2018	34.10
2019	112.45
2020	128.34
Year segment	Slope (*p-value*)
2007-2009	-1.18 (*p=.31*)
2009-2018	2.98 (*p<.001*)
2018-2020	57.69 (*p<.001*)

© 2022 Suk R et al. *JAMA Health Forum.*

eTable 2. Race and ethnicity-specific annual vaccine administration rate and temporal trends among adults aged 27-45 years by sex, 2007-2020

Women	
Non-Hispanic White	
Year	Rate per 100,000 persons
2007	266.27
2008	164.74
2009	110.10
2010	74.21
2011	63.05
2012	57.48
2013	55.32
2014	43.89
2015	46.15
2016	43.88
2017	42.05
2018	57.64
2019	197.01
2020	275.72
Year segment	Slope (*p-value*)
2007-2010	-60.36 (*p<.001*)
2010-2018	-2.13 (*p=.08*)
2018-2020	126.79 (*p<.001*)
Non-Hispanic Black	
Year	Rate per 100,000 persons
2007	178.64
2008	118.51
2009	87.79
2010	59.97
2011	56.59
2012	51.68
2013	51.15
2014	55.76
2015	40.97
2016	45.71
2017	44.57
2018	57.74
2019	195.93
2020	287.26
Year segment	Slope (*p-value*)
2007-2010	-37.33 (*p<.001*)
2010-2018	-0.89 (*p=.38*)
2018-2020	128.84 (*p<.001*)
Hispanic	
Year	Rate per 100,000 persons
2007	201.97
2008	149.58
2009	112.73
2010	81.43
2011	58.20
2012	63.49
2013	54.58
2014	43.21

2015	41.69
2016	46.31
2017	48.74
2018	61.91
2019	209.14
2020	281.52
Year segment	Slope (*p-value*)
2007-2011	-34.63 (*p<.001*)
2011-2018	-0.07 (*p=.97*)
2018-2020	128.03 (*p<.001*)
Asian	
Year	Rate per 100,000 persons
2007	217.72
2008	157.12
2009	113.46
2010	88.34
2011	85.16
2012	71.25
2013	81.69
2014	62.64
2015	70.59
2016	75.06
2017	92.28
2018	111.64
2019	370.28
2020	358.83
Year segment	Slope (*p-value*)
2007-2011	-33.41 (*p=.002*)
2011-2018	4.92 (*p=.08*)
2018-2020	163.95 (*p=.003*)
Unknown	
Year	Rate per 100,000 persons
2007	191.43
2008	116.05
2009	91.58
2010	61.99
2011	56.15
2012	52.10
2013	42.04
2014	46.80
2015	42.93
2016	45.73
2017	37.77
2018	85.49
2019	234.94
2020	277.66
Year segment	Slope (*p-value*)
2007-2009	-55.07 (*p=.06*)
2009-2017	-5.68 (*p=.16*)
2017-2020	85.56 (*p<.001*)

Men
Non-Hispanic White

© 2022 Suk R et al. *JAMA Health Forum.*

Year	Rate per 100,000 persons
2007	4.68
2008	3.69
2009	2.87
2010	6.65
2011	6.90
2012	13.15
2013	12.77
2014	14.04
2015	19.66
2016	22.75
2017	26.42
2018	35.21
2019	113.67
2020	131.71
Year segment	Slope (*p-value*)
2007-2009	-1.14 (*p=.43*)
2009-2018	3.02 (*p<.001*)
2018-2020	59.40 (*p<.001*)
Non-Hispanic Black	
Year	Rate per 100,000 persons
2007	4.55
2008	3.51
2009	2.63
2010	2.79
2011	6.59
2012	14.26
2013	12.17
2014	13.17
2015	17.92
2016	23.02
2017	23.13
2018	30.02
2019	101.28
2020	162.06
Year segment	Slope (*p-value*)
2007-2010	-0.40 (*p=.62*)
2010-2018	3.18 (*p<.001*)
2018-2020	68.86 (*p<.001*)
Hispanic	
Year	Rate per 100,000 persons
2007	4.13
2008	6.57
2009	1.72
2010	6.71
2011	6.59
2012	12.28
2013	15.93
2014	14.62
2015	15.67
2016	17.06
2017	21.20
2018	24.49

© 2022 Suk R et al. *JAMA Health Forum.*

2019	96.09
2020	114.10
Year segment	Slope (*p-value*)
2007-2018	1.93 (*p<.001*)
2018-2020	53.37 (*p=.01*)
Asian	
Year	Rate per 100,000 persons
2007	2.19
2008	4.24
2009	2.21
2010	4.38
2011	5.69
2012	10.43
2013	12.28
2014	17.91
2015	16.91
2016	15.10
2017	34.85
2018	48.66
2019	182.81
2020	131.05
Year segment	Slope (*p-value*)
2007-2017	1.98 (*p=.01*)
2017-2020	44.45 (*p=.02*)
Unknown	
Year	Rate per 100,000 persons
2007	3.78
2008	3.19
2009	1.33
2010	3.93
2011	3.80
2012	15.64
2013	17.31
2014	22.03
2015	13.22
2016	19.22
2017	14.66
2018	34.67
2019	104.55
2020	120.46
Year segment	Slope (*p-value*)
2007-2017	1.00 (*p=.20*)
2017-2020	37.12 (*p<.001*)

© 2022 Suk R et al. *JAMA Health Forum.*

eTable 3. Age distribution of annual HPV vaccination cases among adults aged 27-45 years by sex, 2007-2020

Women						
Year	27 years N (%)	28-30 years N (%)	31-34 years N (%)	35-39 years N (%)	40-45 years N (%)	Total N (100%)
2007	4117 (63.33%)	1167 (17.95%)	554 (8.52%)	368 (5.66%)	295 (4.54%)	6501
2008	2510 (65.45%)	676 (17.63%)	286 (7.46%)	209 (5.45%)	154 (4.02%)	3835
2009	1654 (67.48%)	376 (15.34%)	189 (7.71%)	134 (5.47%)	98 (4.00%)	2451
2010	978 (62.13%)	277 (17.60%)	156 (9.91%)	105 (6.67%)	58 (3.68%)	1574
2011	826 (63.05%)	176 (13.44%)	136 (10.38%)	100 (7.63%)	72 (5.50%)	1310
2012	785 (65.75%)	145 (12.14%)	117 (9.80%)	78 (6.53%)	69 (5.78%)	1194
2013	823 (71.38%)	155 (13.44%)	73 (6.33%)	51 (4.42%)	51 (4.42%)	1153
2014	640 (68.74%)	121 (13.00%)	66 (7.09%)	61 (6.55%)	43 (4.62%)	931
2015	683 (65.99%)	152 (14.69%)	78 (7.54%)	63 (6.09%)	59 (5.70%)	1035
2016	706 (62.70%)	158 (14.03%)	87 (7.73%)	90 (7.99%)	85 (7.55%)	1126
2017	739 (65.81%)	137 (12.20%)	90 (8.01%)	102 (9.08%)	55 (4.90%)	1123
2018	753 (47.18%)	236 (14.79%)	188 (11.78%)	209 (13.10%)	210 (13.16%)	1596
2019	1037 (20.25%)	940 (18.36%)	1081 (21.11%)	1092 (21.32%)	971 (18.96%)	5121
2020	723 (11.87%)	1143 (18.77%)	1403 (23.04%)	1438 (23.62%)	1382 (22.70%)	6089
Men						
Year	27 years N (%)	28-30 years N (%)	31-34 years N (%)	35-39 years N (%)	40-45 years N (%)	Total N (100%)
2007	7 (5.98%)	14 (11.97%)	15 (12.82%)	24 (20.51%)	57 (48.72%)	117
2008	6 (5.94%)	16 (15.84%)	9 (8.91%)	25 (24.75%)	45 (44.55%)	101
2009	6 (10.71%)	14 (25.00%)	5 (8.93%)	11 (19.64%)	20 (35.71%)	56
2010	20 (16.00%)	27 (21.60%)	20 (16.00%)	25 (20.00%)	33 (26.40%)	125
2011	37 (27.21%)	26 (19.12%)	24 (17.65%)	23 (16.91%)	26 (19.12%)	136
2012	114 (41.76%)	45 (16.48%)	39 (14.29%)	36 (13.19%)	39 (14.29%)	273
2013	145 (51.24%)	41 (14.49%)	34 (12.01%)	26 (9.19%)	37 (13.07%)	283
2014	171 (55.16%)	41 (13.23%)	26 (8.39%)	39 (12.58%)	33 (10.65%)	310
2015	212 (49.07%)	77 (17.82%)	56 (12.96%)	40 (9.26%)	47 (10.88%)	432

© 2022 Suk R et al. *JAMA Health Forum.*

2016	266 (49.26%)	103 (19.07%)	64 (11.85%)	54 (10.00%)	53 (9.81%)	540
2017	340 (52.07%)	95 (14.55%)	69 (10.58%)	77 (11.79%)	72 (11.03%)	653
2018	342 (38.00%)	163 (18.11%)	149 (16.56%)	130 (14.44%)	116 (12.89%)	900
2019	483 (16.74%)	609 (21.11%)	682 (23.64%)	638 (22.11%)	473 (16.40%)	2885
2020	369 (12.27%)	603 (20.05%)	723 (24.04%)	730 (24.27%)	583 (19.38%)	3008

© 2022 Suk R et al. *JAMA Health Forum.*

eTable 4. Valent type distribution of annual HPV vaccination cases among adults aged 27-45 years by sex, 2007-2020

Women				
Year	2-valent N (%)	4-valent N (%)	9-valent N (%)	Total N (100%)
2007	0 (0.00%)	6501 (100.00%)	0 (0.00%)	6501
2008	2 (0.05%)	3833 (99.95%)	0 (0.00%)	3835
2009	1 (0.04%)	2450 (99.96%)	0 (0.00%)	2451
2010	26 (1.65%)	1548 (98.35%)	0 (0.00%)	1574
2011	33 (2.52%)	1277 (97.48%)	0 (0.00%)	1310
2012	21 (1.76%)	1173 (98.24%)	0 (0.00%)	1194
2013	14 (1.21%)	1139 (98.79%)	0 (0.00%)	1153
2014	10 (1.07%)	921 (98.93%)	0 (0.00%)	931
2015	7 (0.68%)	884 (85.41%)	144 (13.91%)	1035
2016	7 (0.62%)	455 (40.41%)	664 (58.97%)	1126
2017	3 (0.27%)	222 (19.77%)	898 (79.96%)	1123
2018	3 (0.19%)	187 (11.72%)	1406 (88.10%)	1596
2019	9 (0.18%)	632 (12.34%)	4480 (87.48%)	5121
2020	4 (0.07%)	179 (2.94%)	5906 (96.99%)	6089
Men				
Year	2-valent N (%)	4-valent N (%)	9-valent N (%)	Total N (100%)
2007	0 (0.00%)	117 (100.00%)	0 (0.00%)	117
2008	2 (1.98%)	99 (98.02%)	0 (0.00%)	101
2009	0 (0.00%)	56 (100.00%)	0 (0.00%)	56
2010	3 (2.40%)	122 (97.60%)	0 (0.00%)	125
2011	4 (2.94%)	132 (97.06%)	0 (0.00%)	136
2012	3 (1.10%)	270 (98.90%)	0 (0.00%)	273
2013	1 (0.35%)	282 (99.65%)	0 (0.00%)	283
2014	3 (0.97%)	307 (99.03%)	0 (0.00%)	310
2015	3 (0.69%)	376 (87.04%)	53 (12.27%)	432

© 2022 Suk R et al. *JAMA Health Forum.*

2016	4 (0.74%)	236 (43.70%)	300 (55.56%)	540
2017	2 (0.31%)	138 (21.13%)	513 (78.56%)	653
2018	1 (0.11%)	163 (18.11%)	736 (81.78%)	900
2019	5 (0.17%)	658 (22.81%)	2222 (77.02%)	2885
2020	3 (0.10%)	67 (2.23%)	2938 (97.67%)	3008

© 2022 Suk R et al. *JAMA Health Forum.*

eFigure. Interrupted time series analysis of HPV vaccine administration rates among adults aged 27-45 before and after ACIP recommendation update or FDA approval

a. **Women (ACIP update)**

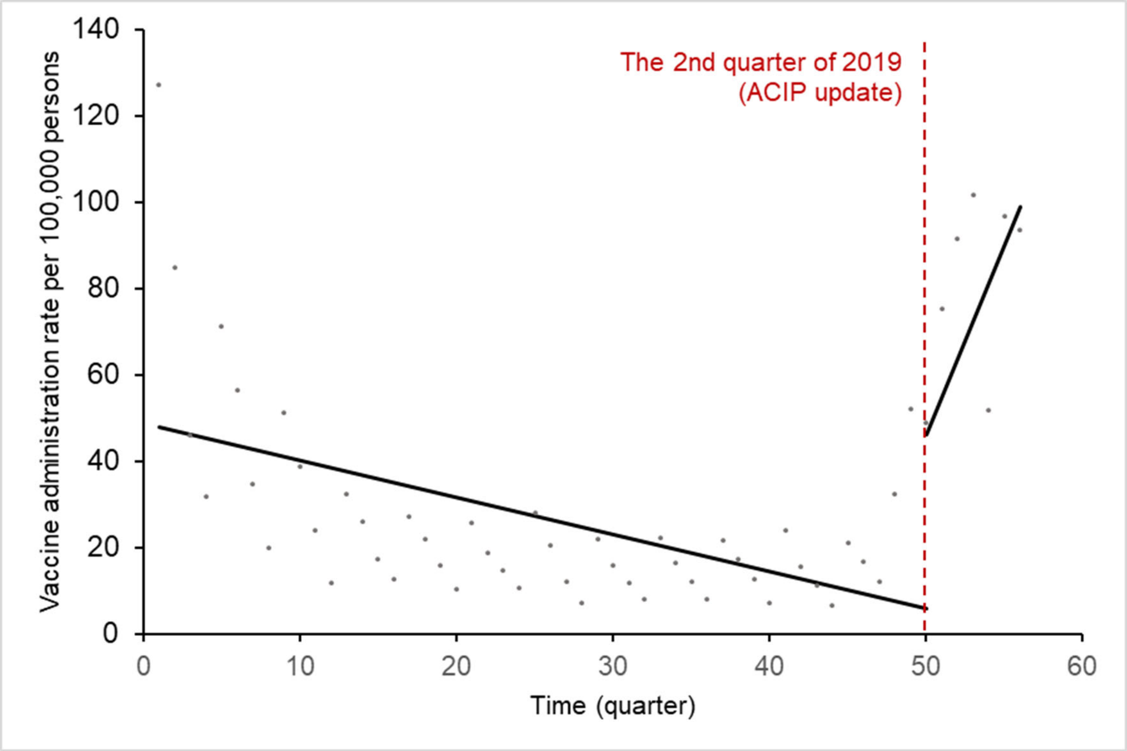

b. **Men (ACIP update)**

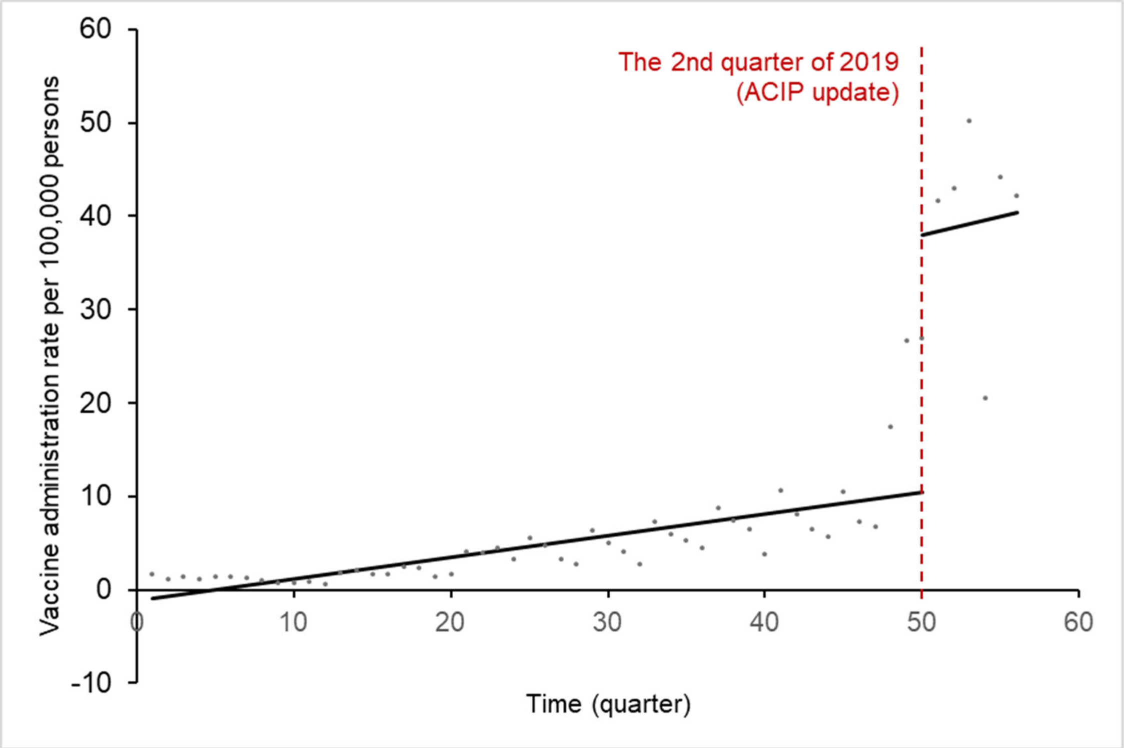

© 2022 Suk R et al. *JAMA Health Forum.*

c. Women (FDA approval)

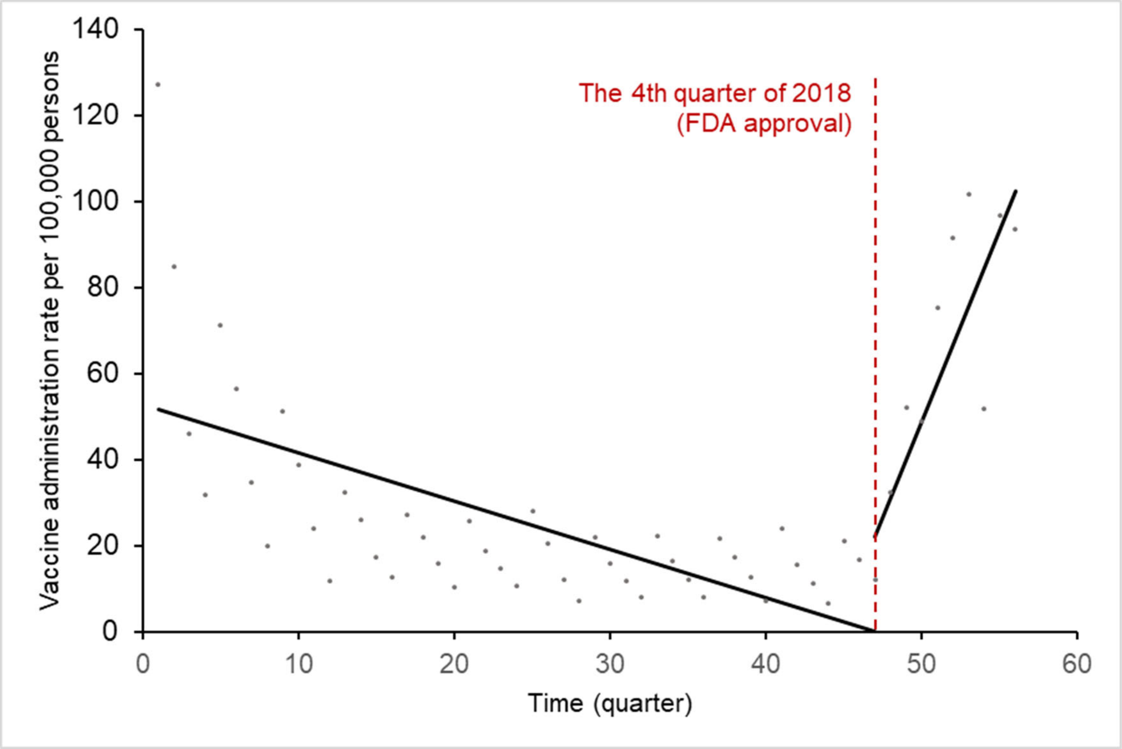

d. Men (FDA approval)

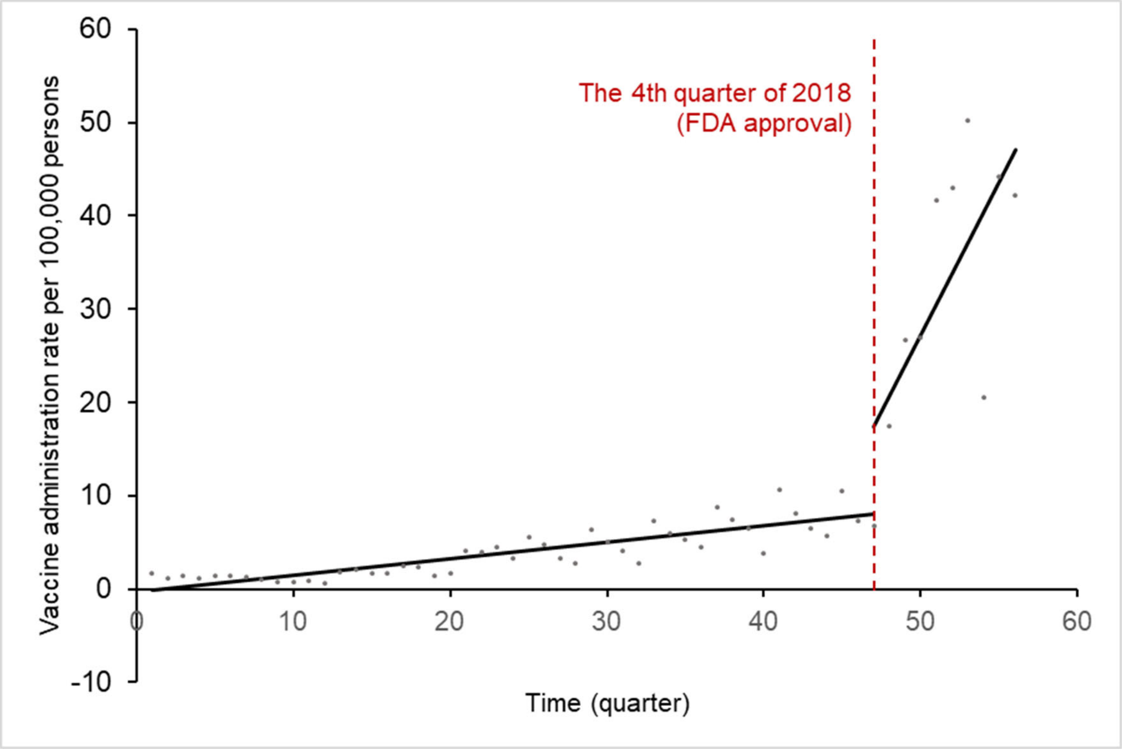

© 2022 Suk R et al. *JAMA Health Forum.*

eTable 5. Sensitivity analysis 1 (annual HPV vaccine administration rate and temporal trends among adults aged 28-45 years by sex) 2007-2020

Women	
Year	Rate per 100,000 persons
2007	90.76
2008	54.47
2009	36.10
2010	28.99
2011	24.21
2012	20.90
2013	16.80
2014	15.34
2015	16.76
2016	18.57
2017	16.92
2018	37.25
2019	183.49
2020	261.96
Year segment	Slope (*p-value*)
2007-2010	-20.58 (*p<.001*)
2010-2018	0.11 (*p=.85*)
2018-2020	132.52 (*p<.001*)
Men	
Year	Rate per 100,000 persons
2007	4.27
2008	3.95
2009	2.31
2010	5.19
2011	4.95
2012	8.02
2013	6.84
2014	6.95
2015	9.88
2016	11.29
2017	12.68
2018	22.36
2019	98.91
2020	118.67
Year segment	Slope (*p-value*)
2007-2018	1.10 (*p<.001*)
2018-2020	60.45 (*p<.001*)

© 2022 Suk R et al. *JAMA Health Forum.*

eTable 6. Sensitivity analysis 2 (annual HPV vaccine administration rate and temporal trends among the birth cohorts who were not eligible before the ACIP change (ages up to 45))

Women	
Year	Rate per 100,000 persons
2007	90.76
2008	42.14
2009	25.15
2010	18.48
2011	16.96
2012	13.69
2013	8.82
2014	9.05
2015	9.39
2016	13.01
2017	9.44
2018	29.04
2019	139.44
2020	204.80
Year segment	Slope (*p-value*)
2007-2009	-33.01 (*p=.003*)
2009-2018	-0.91 (*p=.10*)
2018-2020	108.35 (*p=.003*)
Men	
Year	Rate per 100,000 persons
2007	4.32
2008	3.99
2009	2.46
2010	5.89
2011	6.48
2012	8.02
2013	6.07
2014	6.30
2015	7.76
2016	8.01
2017	10.45
2018	17.59
2019	78.00
2020	95.72
Year segment	Slope (*p-value*)
2007-2018	0.73 (*p<.001*)
2018-2020	49.08 (*p<.001*)

© 2022 Suk R et al. *JAMA Health Forum.*

eTable 7. Sensitivity analysis 3 (annual HPV vaccine administration rate and temporal trends among enrollees with a full year enrollment per every calendar year)

Women	
Year	Rate per 100,000 persons
2007	264.62
2008	163.05
2009	113.84
2010	74.45
2011	62.99
2012	60.03
2013	57.34
2014	50.79
2015	54.67
2016	53.67
2017	50.24
2018	76.45
2019	250.97
2020	339.95
Year segment	Slope (*p-value*)
2007-2010	-60.42 (*p<.001*)
2010-2018	-0.26 (*p=.82*)
2018-2020	154.50 (*p<.001*)
Men	
Year	Rate per 100,000 persons
2007	6.05
2008	4.91
2009	3.1
2010	7.59
2011	7.13
2012	15.02
2013	15.43
2014	16.91
2015	22.91
2016	24.15
2017	29.47
2018	40.29
2019	121.66
2020	157.67
Year segment	Slope (*p-value*)
2007-2009	-1.78 (*p=.32*)
2009-2018	3.45 (*p<.001*)
2018-2020	68.51 (*p<.001*)

© 2022 Suk R et al. *JAMA Health Forum.*

eReferences

1. National Cancer Institute. How Joinpoint Selects the Final Model; https://surveillance.cancer.gov/help/joinpoint/setting-parameters/method-and-parameters-tab/model-selection-method/how-joinpoint-selects-the-final-model. Accessed September 28, 2022.

2. Kim, H.-J., Fay, M.P., Feuer, E.J. and Midthune, D.N. (2000), Permutation tests for joinpoint regression with applications to cancer rates. Statist. Med., 19: 335-351.

3. Linden A. Conducting Interrupted Time-series Analysis for Single- and Multiple-group Comparisons. The Stata Journal. 2015;15(2):480-500.

© 2022 Suk R et al. *JAMA Health Forum.*
